# The cultural context of teaching and learning sexual health care examinations in Japan: a mixed methods case study assessing the use of standardized patient instructors among Japanese family physician trainees of the Shizuoka Family Medicine Program

**DOI:** 10.1186/s12930-015-0025-4

**Published:** 2015-10-07

**Authors:** Cameron G. Shultz, Michael S. Chu, Ayaka Yajima, Eric P. Skye, Kiyoshi Sano, Machiko Inoue, Tsukasa Tsuda, Michael D. Fetters

**Affiliations:** Department of Family Medicine, University of Michigan, 1018 Fuller Street, Ann Arbor, MI 48104-1213 USA; University of Michigan Medical School, Ann Arbor, MI USA; Virginia Mason Medical Center, Internal Medicine Residency, Seattle, Washington USA; Department of Family Medicine, Tokushukai Hospital Corporation, Haibara General Hospital, Makinohara, Shizuoka Japan; Department of Family and Community Medicine, Hamamatsu University School of Medicine, Hamamatsu, Shizuoka Japan; Akatchi Family Medicine Center, Kikugawa, Shizuoka Japan

**Keywords:** Standardized patient instructors, Japan, Family medicine, Sexual health

## Abstract

**Background:**

In contrast to many western nations where family medicine is a cornerstone of the primary care workforce, in Japan the specialty is still developing. A number of services within the bailiwick of family medicine have yet to be fully incorporated into Japanese family medicine training programs, especially those associated with sexual health. This gap constitutes a lost opportunity for addressing sexual health-related conditions, including cancer prevention, diagnosis, and treatment. In this mixed methods case study we investigated the perceived acceptability and impact of a standardized patient instructor (SPI) program that trained Japanese family medicine residents in female breast, pelvic, male genital, and prostate examinations.

**Case description:**

Building on an existing partnership between the University of Michigan, USA, and the Shizuoka Family Medicine Program, Japan, Japanese family medicine residents received SPI-based training in female breast, pelvic, male genital, and prostate examinations at the University of Michigan. A mixed methods case study targeting residents, trainers, and staff was employed using post-training feedback, semi-structured interviews, and web-based questionnaire.

**Discussion and evaluation:**

Residents’ and SPIs’ perceptions of the training were universally positive, with SPIs observing a positive effect on residents’ knowledge, confidence, and skill. SPIs found specific instruction-related approaches to be particularly helpful, such as the positioning of the interpreter and the timing of interpreter use. SPIs provided an important opportunity for residents to learn about the patient’s perspective and to practice newly learned skills. Respondents noted a general preference for gender concordance when providing gender-specific health care; also noted were too few opportunities to practice skills after returning to Japan. For cultural reasons, both residents and staff deemed it would be difficult to implement a similar SPI-based program within Japan.

**Conclusions:**

While the SPI program was perceived favorably, without sufficient practice and supervision the skills acquired by residents during the training may not be fully retained. Deep-rooted taboos surrounding gender-specific health care appear to be a significant barrier preventing experimentation with SPI-based sexual health training in Japan. The feasibility of implementing a similar training program within Japan remains uncertain. More research is needed to understand challenges and how they can be overcome.

## Background

In contrast to many western nations where family medicine is a cornerstone of the primary care workforce, in Japan the specialty is still developing [1–5]. A number of services within the bailiwick of family medicine have yet to be fully incorporated into Japanese family medicine training programs, especially those associated with sexual health [6]. This gap contributes to a lost opportunity for identifying and treating sexual health-related conditions, as well as cancer prevention, early diagnosis, and treatment.

To address this gap, the Shizuoka Family Medicine Program, Japan, partnered with the University of Michigan Department of Family Medicine, United States of America (USA), to implement a standardized patient instructor (SPI) program to provide Japanese family medicine residents with training in female breast, pelvic, male genital, and prostate examinations at the University of Michigan. As part of a larger collaborative educational project—the Shizuoka-University of Michigan Advanced Residency Training, Education and Research in Family Medicine (SMARTER-FM) [7]—the SPI program was a key component of providing visiting residents with hands-on, real life experience in performing these exams.

### Epidemiological background

As shown in Table 1, incidence of prostate, breast (female only), uterus, cervical, and ovarian cancers in Japan are markedly higher than the global average. Cervical and mammography screening in Japan is much lower than that of other developed nations [8]. While Japanese surveillance data on sexually transmitted infections (STIs) are incomplete [9], evidence suggests rates of some STIs may be high [9–12]. Human papillomavirus (HPV) infection, for example, may be common among some segments of the population, particularly women of reproductive age [10, 13, 14].

**Table 1:** Health indicators related to breast, pelvic, male genital, and prostate examinations, and the primary care workforce

	World	Japan
Age-standardized incidence rate for cancer per 100,000 population, 2010 [37]		
Prostate	37.9	56.0^a^
Breast (female only)	60.8	78.4^a^
Uterus	22.0	28.1^a^
Cervix uteri	11.2	14.2^a^
Corpus uteri	10.4	13.5^a^
Ovary	9.0	11.3^a^
Estimated number of new cancer cases attributable to HPV infection, 2008 [11]	610,000	11,000
Herpes simplex virus type 2, population aged 15–49 years, prevalence in millions [38]	535.5	4.8
Cervical cancer screening, percentage women screened aged 20–69 years, 2009 [8]	85.9 (United States) 78.7 (United Kingdom) 38.9 (Mexico)^b^	24.5
Mammography screening, percentage of women aged 50–69 years screened, 2009 [8]	81.1 (United States) 74.0 (United Kingdom) 16.6 (Mexico)	23.8
Practicing medical doctors per 1000 population, 2009 [8]	2.4 (United States) 2.7 (United Kingdom) 2.0 (Mexico)	2.2
General practitioners, as a share (%) of total medical doctors, 2009	12.3 (United States) [8]^c^ 27.1 (United Kingdom) [39]^d^ 36.7 (Mexico) [8]^c^	16.7–34.4 [39]^d,e^
Doctor visits per year [18]	3.9 (United States) 5.9 (United Kingdom) 2.8 (Mexico)	13.2

*HPV* human papillomavirus

^a^Based on Japan model population in 1985

^b^Based on data from 2000

^c^Based on data from 2009

^d^Based on data from 2004

^e^The primary care workforce in Japan includes many physician types, including internal medicine (41.8 %), ophthalmology (8.0 %), orthopedics (7.2 %), pediatrics (6.8 %), otolaryngology (5.6 %), surgery (5.5 %), obstetrics/gynecology (4.7 %), dermatology (4.7 %), and others (15.6 %)

### Health care in Japan

Like most developed nations, Japan provides its citizens with universal health coverage. Implemented in 1961, Japan’s national insurance program combines employee- and community-based plans. Fees charged by hospitals and physicians are regulated, and most citizens have a co-payment rate of 30 % [15]. The primary and specialty care disciplines in Japan are not wholly distinct [16, 17], and the training and services provided by primary health care physicians can vary from clinic to clinic, and from physician to physician [1–3, 16].

As measured by the number of doctor visits per year, Japanese are among the top consumers of health care in the world (Table 1). An important driver of Japanese health care utilization stems from the absence of a gatekeeping mechanism—any patient can essentially drop in at any clinic at any time, and outpatient specialty and hospital care do not generally require referrals [16]. In addition, Japan’s fee structure encourages more frequent, shorter visits [18]. Despite excellent access in this system, health care is often fragmented, and many gender-specific services (e.g., female breast, pelvic, male genital, and prostate examinations) have yet to be fully incorporated and accepted into Japanese family physicians’ scope of care [6].

In 2004, an obligatory 2-year postgraduate training program (*shoki kenshū*) was instituted for all new medical school graduates. The focus of this training is on general medicine, with its curriculum centered on hospital-based general internal medicine, general surgery, emergency medicine, anesthesiology, pediatrics, obstetrics and gynecology, psychiatry, and community medicine [19]. As part of the advanced training period (*kōki kenshū*), the Japanese family medicine residency lasts 3 years. Residency programs in family medicine remain inconsistent across training sites [2, 3, 20], and qualified trainers remain in short supply [3, 21, 22].

### Sex, taboo, and gender-specific health care

Modesty and masculinity—central to gender identity in many cultures—can have a large impact on how health care is perceived and utilized [23, 24]. In Japanese culture, Western-style medicine may at times be perceived as invasive, especially for procedures requiring the patient to undress [24]. Some Japanese believe that discussions about genitalia and the gendered, sexualized body violate a “code of *civilized morality*” [25]. According to this code, sex-related behaviors outside of marriage are thought to be indecent and require “silence or euphemism” [25]. Hence, for those influenced by this code, the act of seeking gender-specific health care may in and of itself be intensely embarrassing.

#### Gender-specific health care for women

Examination rooms for general and gynecological care in Japan are typically not private, constructed with thin partitions (often open on one end), and may or may not have a curtain (in lieu of a door) [26]. With the intent of preserving modesty, gynecological exam rooms may have a curtain hiding the woman’s upper body and face [26, 27]. For many Japanese women this clinical setting is not acceptable, and can contribute to delaying or foregoing basic gynecological care. For example, female Japanese university students reported fear and embarrassment as principal reasons for avoiding gynecological care [28], and among Japanese women of reproductive age experiencing unusual menstrual symptoms, nearly one-fifth cited “feeling resistance or aversion to gynecologists and hospitals” as a contributing factor in their decision to not seek care [29].

Despite having more health care consultations per year when compared to most other countries, some Japanese women report access-related problems for contraceptive care. One consequence of the poor access is that abortion may at times become a default method of birth control [30]. Evidence suggests Japanese women are more likely to face barriers in securing modern contraception when compared to women in the USA and France; moreover, Japanese women are less likely to understand the non-contraceptive benefits of oral contraceptives [31]. This finding is supported by family planning indicators that show Japanese women fall far behind women from other developed countries in terms of modern contraception use: the proportion of married or in-union women aged 15–49 years using any modern method of contraception in 2013 is 50 % for Japan, 70 % for the USA, 72 % for France, and 81 % for the United Kingdom [32]. One explanation for the lackluster uptake may stem from limited knowledge about oral contraceptives among both patients and physicians [33].

#### Gender-specific health care for men

Although Northeastern Asia has an extremely heterogeneous culture, some cultural norms are shared across geo-political borders and emerge as cultural themes. Like men from many cultural groups, Asian men may at times act in ways to preserve a sense of masculinity. Identity-related attributes endorsed by many Asian men include “having an active sex life,” “having success with women,” and “avoiding shameful situations.” Among men in Japan, the attributes of honor and control are also strongly endorsed [23]. When considering these attributes as part of a cultural schema or meme, they likely play a role in Japanese men’s hesitancy to seek (and physicians’ reluctance to provide) the male genital exam, prostate exam, or other service that might threaten men’s sense of vitality, honor, or control.

### The Shizuoka Family Medicine Program

The Shizuoka Family Medicine Program, located in Shizuoka prefecture, Japan, has two main clinical sites located approximately 32 km (20 miles) apart: Kikugawa (population: 47,000) and Mori-machi (population: 19,000). Since its establishment in 2010, the Shizuoka Family Medicine residency program has had class sizes ranging from 1 to 6 residents per year. Residents alternate between clinics for outpatient experiences and rotations at three local hospitals (Kikugawa, Mori-Machi, and Iwata). While the Shizuoka Family Medicine residency program trains residents to provide care across the life span—from cradle to grave—the program has no on-site or domestic SPI program to provide training in female breast, pelvic, male genital, and prostate examinations. Similar to other family medicine residency programs in Japan, training for these exams is limited to practice with manikins or instruction with actual patients.

## Case description

The purpose of this mixed methods case study was twofold: to investigate the SPIs’ and Japanese residents’ perceptions about the training experience in the USA, and to examine the perceived impact and acceptability of performing the learned skills from residents and other key informants after residents returned to Japan.

### Standardized patient instructor-based training at the University of Michigan

**Fig. 1:**
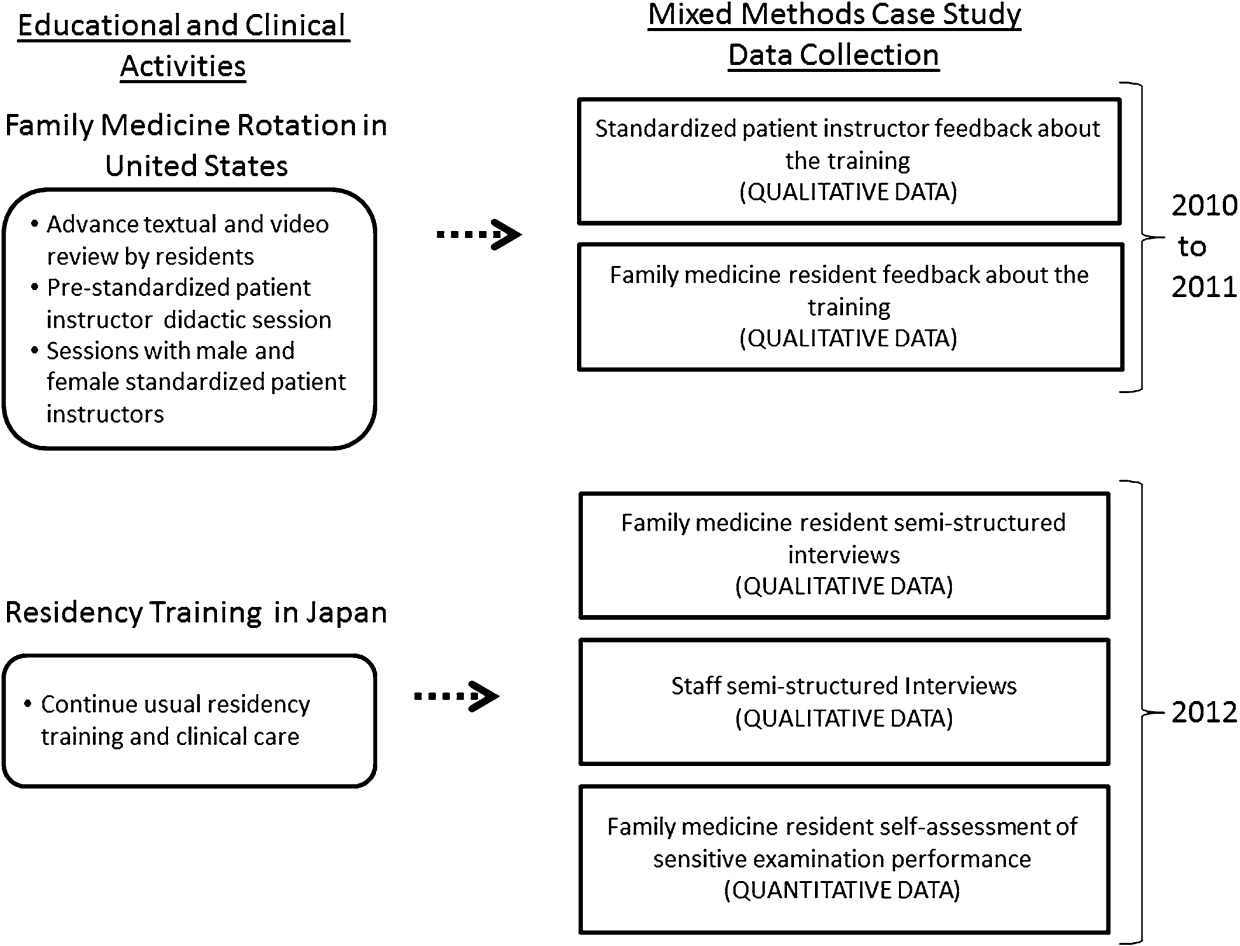
Overview of the standardized patient instructor experience for Japanese family medicine residents and the mixed methods case study procedures

As part of the SMARTER-FM project, all Shizuoka Family Medicine residents had a 2-week rotation at the University of Michigan, usually in their first year (Fig. 1). In addition to other clinical teaching and experiences during this rotation, residents received SPI-based training in female breast, pelvic, male genital, and prostate examinations. The SPI-based training started with residents reviewing in advance textual materials and online videos, followed by didactic sessions—delivered by an attending family medicine physician from the University of Michigan (EPS)—focusing on anatomy and proper examination technique. Didactic sessions were augmented with hands-on practice using models simulating physical examination findings. On separate days, residents worked with a female SPI for female breast and pelvic exams, and a male SPI for male genital and prostate exams. An experienced Japanese-English interpreter was present for each resident throughout the training. Ample opportunity was provided for one-on-one practice and feedback. During the 2-week experience, residents worked closely with University of Michigan family medicine faculty at the Japanese Family Health Program in Ann Arbor, MI (USA), where the SPI-based training was reinforced through focused instruction with consenting patients.

### Design

This mixed methods case study was reviewed and classified as exempt by the University of Michigan Institutional Review Board. As illustrated in Fig. 1, data were collected at three time periods. Years 1 (2010) and 2 (2011) indicate the first and second wave of Shizuoka Family Medicine residents, respectively, receiving the SPI-based training at the University of Michigan. Year 3 (2012) indicates the follow-up data collection period in Japan (i.e., 2 years post-training for wave 1 residents, and 1 year post-training for wave 2 residents).

The study included four data collection arms: (1) post-training evaluations from Shizuoka Family Medicine residents and SPI instructors (years 1 and 2); (2) follow-up semi-structured interviews with Shizuoka Family Medicine residents (year 3); (3) semi-structured interviews with key informants (nurses and medical assistants) from Shizuoka Family Medicine (year 3); and (4) a web-based questionnaire targeting Shizuoka Family Medicine residents (year 3).

### Arm 1: post-training evaluations

Written feedback about the training was solicited from both Shizuoka Family Medicine residents and SPIs. Administered upon completion of the 2-week rotations at the University of Michigan (and for residents, before returning to Japan), participants were asked to provide information about their overall experience and reflections on the SPI exercises. Resident and SPI evaluations were completed in Japanese and English, respectively.

### Arm 2: follow-up semi-structured interviews with residents

The semi-structured interview guide was developed by the research team using an iterative, consensus-based process, wherein study investigators reviewed and revised the guide to ensure it was both easy to use and adequately captured the topics of interest. The guide was designed to elicit residents’ perspectives on several domains: (1) the provision of gender-specific health care in the Japanese family medicine setting; (2) physician and patient comfort in performing gender-specific examinations; (3) impact and utility of the SPI-based training at the University of Michigan; and (4) the feasibility of implementing a similar training program in Japan.

Japanese residents who completed the University of Michigan SPI-based training were invited to participate by a research assistant (MSC) trained in qualitative interviewing. All interviews were conducted in-person and in Japanese. Individual interviews were scheduled at a time agreeable to the resident, and conducted in a location offering privacy. Subjects provided verbal consent prior to the interview.

### Arm 3: semi-structured interviews with Shizuoka Family Medicine nurses and medical assistant staff

The interview guide developed for Arm 2 was modified for use with nurses and medical assistants to address parallel content. It sought information on resident performance in providing gender-specific health care, and the perceived impact of the SPI training. Using a purposive sampling strategy, nurses and medical assistants who worked closely with Shizuoka Family Medicine residents were invited to participate. Interview procedures for these key informants mirrored that of the residents, including administration by the same research assistant (MSC).

### Arm 4: Web-based questionnaire targeting Shizuoka Family Medicine residents

The web-based questionnaire was developed by the research team using an iterative, consensus-based process to parallel and supplement the qualitative data collection. The primary focus of the instrument was residents’ self-perceived experience and proficiency for each of the examinations. Questionnaires were completed at a time and location of the residents’ choosing.

### Qualitative, quantitative, and mixed methods analyses

The qualitative feedback data from Arm 1 were organized into a matrix constituting 30 pages of single spaced text. This allowed parallel comparison of resident and SPI feedback. Two bilingual and experienced qualitative researchers (MSC and AY) immersed themselves in the feedback data, and used an editing approach to reduce the data into salient themes (Table 2) [34]. Due to space constraints and to simplify interpretation, example quotations for each theme are not presented; rather, we provide summative descriptive statements [35].

**Table 2:** Summary of the written qualitative feedback of family medicine residents and standardized patient instructors collected immediately after training sessions

Stage	Resident comments n = 8	SPI comments n = 2
Overall	Wonderful experience Felt like I advanced more than any other teaching Reviewing anatomy, having didactic, then performing Learning directly from the patient, instead of books, videos, and observing senior physicians Teaching systematic, better than during clinical care	Went extremely well Enjoyed teaching, learned from experience with non-English speakers Met expectations for being polite, gracious Pleasantly surprised by curiosity, desire to clarify and ask questions Discovered resident learning experiences in Japan mostly had been passive, observational Some learners initially tentative Agreed to being photographed after the teaching session
Pre-SPI encounter	Observing examinations in the clinic prior to SPI experience made it more effective Helpful to review online written materials & videos on anatomy, and how to perform examinations	NA: SPI were not asked to provide
Pre-SPI lecture/coaching	Learning how to examine using manikin models Learning the procedures for interacting with an SPI	NA: Provided by faculty member
SPI session	SPI comfortable with teaching SPI demonstrating how to do exam, then doing it SPI knew own physical findings, and showed them Understanding the patient’s perspective (e.g., anxiety, discomfort, modesty) Individualized teaching in detail, in person Learner repeating over and over until got it right (e.g., finding cervix with speculum) Pacing the teaching to the learner’s ability Appreciation of teaching from the patient’s perspective about modesty, protecting it Learning different patterns of examination Feeling a real lump	Focused on “reading, watching, doing” Defined scope of session: e.g., procedures, role of SPI Encouraged questions “Cheat sheet”—SPI prepared, helped learner Inquiring about learners’ previous examination experiences Taught examination techniques, communication skills, sequence of the examination, putting the patient at ease, when to use chaperone, accommodating family members, positioning (e.g., common patient preferences, and accommodating co-morbidities) Teaching how to protect patient modesty, how to incorporate genitourinary exam routinely or focused into overall examination SPIs excited when learner palpated actual findings Enthusiasm of learners made session longer than SPI expected
Using interpreter	Having an interpreter present helpful to understand (pre-session) Very helpful for understanding and clarification (during SPI session)	Using an interpreter was novel Reading in advance about how to use interpreter Took nearly twice as long using interpreter Interpreter used first person Tried speaking initially in phrases, but interpreter preferred full sentences Positioned interpreter facing away, toward wall during examination, or caudad to exposed genitalia (male SPI on female interpreter) After getting used to interpreter, became easier, flowed better When learner practiced combining examination skills and communication to patient, opted to NOT use interpreter to facilitate the learner naturally integrating examination and communication skills (rather than disrupting flow by using interpreter)
Improvements	United States speculum different from Japan; not used to it Feel he/she needs to train many times after the session by oneself Video recording of the teaching session for reference for self-study would be helpful Want to confirm if performing examinations could be done by oneself Need manikin models with abnormal findings	

*SPI* standardized patient instructor

Qualitative interviews from Arms 2 and 3 were digitally recorded, and the recordings transcribed verbatim in Japanese. Interviews produced a total of 228 pages of single-spaced, Japanese text. Under the supervision of an experienced researcher (MDF), interview data were analyzed by the same team members (MSC and AY) who initially immersed themselves in the feedback data. A coding scheme was developed using an iterative, consensus-based process [36]. Data were further reduced into salient themes, and a matrix was developed to illustrate comparison between resident and SPI feedback, and resident and ancillary staff interviews. To show the breadth and relative frequency of comments, final categories were transformed into quantitative data by counting the number of respondents who endorsed a given theme. This analysis was designed to understand how the qualitatively elicited opinions about specific topics were distributed among the three groups. All text material was analyzed qualitatively in the language of collection (Japanese or English) as each analyst (MDF, MSC, and AY) is fluent in both languages. Summary statistics were analyzed descriptively for the quantitative data produced by the web-based survey in Arm 4. By looking across qualitative and quantitative findings, we examined the extent to which findings from each arm corroborated (or contradicted) each other.

## Discussion and evaluation

Three Shizuoka Family Medicine residents participated in the SPI-based training in year 1, and 6 participated in year 2. Eight residents provided post-training feedback. Male (n = 1) and female (n = 1) SPI instructors provided feedback from the perspective of the teacher. In Japan, all 9 residents participated in the follow-up interview and completed the web-based questionnaire. In addition, seven key informants—5 nurses and 2 medical assistants—participated in interviews.

Four overarching themes were identified: (1) experience with the SPI training program; (2) perceived proficiency in performing female breast, pelvic, male genital, and prostate examinations; (3) gender concordance between patients and residents; and (4) women’s and men’s health issues.

### Experience with the SPI training program

A summary of the comments expressed by residents and SPIs following the SPI training experience is presented in Table 2. Corroborative information and salient themes from semi-structured interviews (arms 2 and 3) are outlined in Table 3.

**Table 3:** Resident, nurse, and medical assistant reports during semistructured interviews regarding skill proficiency, relevance of gender, sexual health discussions, and potential for SPIs in Japan

Topic	Residents in year 1 (n = 6)	Residents in year 2 (n = 3)	Nurses and medical assistants (n = 7)
Examination proficiency			
Have performed pelvic exams many times	3	1	–
Unsure if able to find abnormalities/diagnose in pelvic exams	4	1	–
Does not get to perform breast exams often	5	2	–
Does not get to perform male genital exams often	6	3	–
Patient(s) seemed uncomfortable during male genital exam	2	1	–
Can properly feel the prostate during digital rectal exam	4	3	–
Gender concordance/discordance			
No issues with gender concordance	4	2	7
No issues with gender discordance	1	1	2
Prefers gender match	2	1	3
Female patients tend to request female physicians	1	1	5
Difficult to talk about sexual health when gender discordant	4	1	3
Able to ask appropriate questions regardless of concordance	1	–	3
Women’s and men’s health			
Discusses sexual health and vaccinations with female patients	3	1	3
Recommends contraception for female patients	3	–	2
Recommends pap smears for female patients	3	2	–
Recommends smoking cessation outpatient services for male patients	–	2	3
Cannot think of any issues specific to men’s health	1	–	3
Should improve on screening male patients for erectile dysfunction	2	2	–
SPI training			
It was a great experience	3	2	–
Allows for learning that would not otherwise be possible in Japan	2	2	–
Would prefer more practice either at University of Michigan or in Japan	4	1	–
Would be difficult to have in Japan	2	–	4
Would be difficult to find people willing to become SPIs	2	1	1
Would like to have an SPI program in Japan	5	3	3

*SPI* standardized patient instructor

Resident and SPI feedback about the SPI-based training was universally positive, with both residents and SPIs praising all aspects of the training including the pre-session studies, didactics with hands on teaching, and the SPI teaching encounters. Resident and SPIs alike identified several components of the training as critically essential, including learning about communication skills, practicing of psychomotor skills, identifying actual findings during the examination, and receiving feedback. Residents and SPIs noted the utility of the interpreter for mutual understanding, though this did raise some new challenges for the SPIs. Residents described that family medicine residency programs in Japan do not utilize SPIs for physical examinations or procedures.

One to two years after their return to Japan, residents continued to highly value their SPI training experience. Several explanations were provided for the sustained satisfaction, including an appreciation of the training’s focus to improve both interpersonal and clinical skill; specifically, it was noted that the training provided an opportunity to learn directly from the patient and about the patients’ perspective, and it helped residents learn how to perform the examinations while also maintaining the patient’s comfort and dignity. The training was also described as an excellent stepping-stone for obstetrics/gynecology and urology rotations. Residents noted that the training made a positive difference in their ability to practice medicine more generally, as it provided skills in how to be more sensitive to patients’ needs. Nurses and medical assistants noted improved patient care by residents after the SPI-based training, though they did not necessarily consider the uptick in performance to be directly related to the training.

Despite participants’ positive experience with the SPI training, there was little optimism about the potential for such training to take hold within Japan (Table 3). Numerous cultural and social barriers were reported, the most significant of which were perceived challenges to recruiting Japanese SPIs. Study participants indicated the potential for volunteers to be stigmatized if their identity were leaked to the community. Japanese identity was also mentioned as a barrier, noting that Japanese are easily embarrassed and care a great deal about how they are perceived by peers. Before use of SPIs could become widespread in Japan, it was suggested that the general public would first have to recognize and understand the value of SPIs to medical education. It was also noted that explicit support from a credible social institution (e.g., the government) would likely be necessary before an SPI-based training program could be sustained.

### Perceived proficiency in performing female breast, pelvic, male genital, and prostate examinations

As indicated in Table 4, findings from the web-based questionnaire show that residents’ experience in performing female breast, pelvic, male genital, and prostate examinations varied widely. With the exception of the pelvic examination, residents’ experience with performing the examinations was very limited. The count for female breast and male genital examinations was particularly low, with some residents having never performed them. These data corroborate the residents’ reports about the difficulty of continuing these exams in Japan and the limited opportunity to practice their newly acquired skills.

**Table 4:** Self-reported estimates of the number of examinations performed by Shizuoka family medicine residents, from resident questionnaires (n = 9)

Examination	Range	Mean	Median	Standard deviation
Women’s health				
Breast exam	0–20	6	5	6
Pelvic exam	15–600	198	100	218
Men’s health				
Genital exam	0–40	8	1	15
Prostate exam	4–30	14	7	11

Several residents noted that some male patients have had a negative response to the male genital exam. More than half of residents expressed that although they knew how to perform a pelvic exam, they were uncertain if they could properly identify abnormalities or make diagnoses on their own (Table 3).

### Gender concordance between patients and residents

Despite the majority of interview participants indicating that gender concordance between patients and physicians had little impact on care, more than one-third described that exams went more smoothly when pairs were gender concordant; moreover, nearly half noted having difficulty in discussing topics related to sexual health in gender discordant pairs (Table 3). Improved communication and decreased embarrassment were reported as the principle benefits of concordance, particularly for younger female patients who some participants described as more likely to request a female physician. It was noted that the Shizuoka Family Medicine program’s administration preferred gender concordance during encounters where sexual health was the primary reason for the health care visit. Participants also described that reception staff and nurses commonly assigned specific residents to specific patients based on gender and the patient’s chief complaint.

### Women’s health issues

The majority of participants stated that residents do an adequate job of discussing sexual health when working with female patients (e.g., contraception, screening, and menstrual cycles/menopause), even if the patient’s chief complaint was unrelated to sexual health. Areas noted as needing improvement included taking steps to preserve patients’ comfort (e.g., not leaving patients in an exposed or uncomfortable position while the resident is seeking help from an attending), being sensitive to patients who lack comfort in discussing sexual health (e.g., being careful to not overwhelm the patient with questions related to sexual health, especially if the patient is sick), and increasing vaccination rates (e.g., for HPV).

### Men’s health issues

Participants’ recognition of men’s health issues was almost exclusively limited to prostate and/or urinary problems. Participants rarely raised the topics of erectile dysfunction and sexually transmitted infections. For erectile dysfunction, it was noted that the topic was generally not discussed with patients unless the patient first raised the issue on their own. Other men’s health issues described as needing to be better addressed included education on contraceptive methods and discussing sexual health.

## Conclusions

To our knowledge, the University of Michigan SPI-based training program for Japanese family medicine residents is the only one of its kind. In the absence of this training, practical experience for Shizuoka Family Medicine residents in female breast, pelvic, male genital, and prostate examinations would have been almost entirely limited to core obstetrics/gynecology and urology rotations in affiliated settings. While the use of SPIs to assist with training has many potential benefits, their use within Japan challenges long-standing and strongly-held sociocultural beliefs about gender, identity, and sex. To overcome such deeply engrained beliefs will likely require considerable effort, and may necessitate securing support from the local community and respected institutions (e.g., medical schools, professional organizations, government). Cultural taboos notwithstanding, findings from this evaluation demonstrate the feasibility of implementing an SPI-based training program, that the skills learned were transferable to the practice of family medicine in Japan, and that such a program is both acceptable and viewed favorably by key stakeholders.

There is a bit of a chicken and egg phenomenon relative to the incorporation of sexual health into the practice of family medicine in Japan. While health indices in relevant diseases (e.g., sexually transmitted infections, cancer) need interventions, there are few faculty trained in how to provide this care. Since there is little comfort providing the care, few examinations are actually performed and the care is not routine. Consequently, the care does not seem routine to patients, and the services are not sought by patients. Our hope was that the SPI training would help to break this cycle within the Shizuoka Family Medicine program in Japan, but our efforts achieved only limited success. This experience illustrates what we believe is a common problem when family medicine is adopted in cultures with very little history of addressing women’s and men’s health as part of routine primary care. As for how to further break the cycle, one possibility is much stronger self-promotion by trained family physicians themselves during individual patient consultations, such as raising sexual health and cancer prevention care during routine visits. A second possibility is for the practice to more strongly educate the patient population about sexual health services that are available. Doing so will necessitate accommodating (and in some cases overcoming) very strong traditions and taboos. And as articulated by study participants, sustained change may require explicit sanction from institutions already possessing the public’s trust.

This research has several limitations. First, the SPI training occurred in a single training site (the University of Michigan), and follow-up was confined to a single, relatively new family medicine residency program located in two geographic areas (Kikugawa and Mori-Machi). Findings should be interpreted cautiously, as they may not be generalizable to all Japanese medical training environments. Second, the number of residents participating in the SPI-based training was small. While all participants described the training favorably, it is possible that others could have a less positive experience. Follow-up studies using a more diverse resident population are needed. Third, the duration between training and follow-up interviews ranged from 1 to 2 years; this difference accounts for some of the variation in the number of examinations performed. Fourth, evaluation methods relied on respondents’ self-reported perceptions and experiences. It is possible that participants may have underreported or overreported the impact of the training on their performance and skill. To combat this limitation, future research could measure the impact of SPI-based training on objectively-derived measures, such as correct diagnoses or correct adherence to an examinations’ ordered steps. And last, given deep-rooted taboos surrounding gender-specific health care (e.g., code of civilized morality), study participants may have been influenced (knowingly or unknowingly) by their own cultural biases. Given the study’s mixed methods design and the application of multiple data collection procedures—using post training feedback forms, semi-structured interviews, and a web-based questionnaire—it is our hope that the potential impact of such biases were appreciably reduced.

While the University of Michigan SPI program was perceived favorably, the feasibility and sustainability of implementing a similar program within Japan remains uncertain. Deep-rooted taboos surrounding gender-specific health care appear to be a significant barrier. Without sufficient practice, supervision, and promotion of the sexual health services offered by family physicians, skills acquired by residents during SPI-based training may not be adequately retained or applied over time. More research is needed to understand these challenges, including how they can be overcome to improve women’s and men’s health in Japan.

## Abbreviations

AY: Ayaka Yajima
EPS: Eric P. Skye
HPV: human papillomavirus
MDF: Michael D. Fetters
MSC: Michael S. Chu
SPI: standardized patient instructor
STIs: sexual transmitted infections
SMARTER-FM: Shizuoka-University of Michigan Advanced Residency Training, Education and Research in Family Medicine
USA: United States of America
